# Protective Effects and Mechanisms of Esculetin against H_2_O_2_-Induced Oxidative Stress, Apoptosis, and Pyroptosis in Human Hepatoma HepG2 Cells

**DOI:** 10.3390/molecules29071415

**Published:** 2024-03-22

**Authors:** Ying Luo, Tenglong Chang, Shiting Huang, Jing Xiang, Shuangyang Tang, Haiyan Shen

**Affiliations:** The Institute of Biochemistry and Molecular Biology, Hunan Provincial Key Laboratory for Special Pathogens Prevention and Control, Hengyang Medical College, University of South China, Hengyang 421001, China; luoyingps@163.com (Y.L.); changtl168@163.com (T.C.); shea1119@163.com (S.H.); xxj19980705@163.com (J.X.); tsy7909@126.com (S.T.)

**Keywords:** esculetin, oxidative stress, apoptosis, pyroptosis, JNK, liver diseases

## Abstract

Oxidative stress plays a crucial role in the pathogenesis of many diseases. Esculetin is a natural coumarin compound with good antioxidant and anti-inflammatory properties. However, whether esculetin can protect HepG2 cells through inhibiting H_2_O_2_-induced apoptosis and pyroptosis is still ambiguous. Therefore, this study aimed to investigate the protective effects and mechanisms of esculetin against oxidative stress-induced cell damage in HepG2 cells. The results of this study demonstrate that pretreatment with esculetin could significantly improve the decrease in cell viability induced by H_2_O_2_ and reduce intracellular ROS levels. Esculetin not only apparently reduced the apoptotic rates and prevented MMP loss, but also markedly decreased cleaved-Caspase-3, cleaved-PARP, pro-apoptotic protein (Bax), and MMP-related protein (Cyt-c) expression, and increased anti-apoptotic protein (Bcl-2) expression in H_2_O_2_-induced HepG2 cells. Meanwhile, esculetin also remarkably reduced the level of LDH and decreased the expression of the pyroptosis-related proteins NLRP3, cleaved-Caspase-1, Il-1β, and GSDMD-N. Furthermore, esculetin pretreatment evidently downregulated the protein expression of p-JNK, p-c-Fos, and p-c-Jun. Additionally, anisomycin, a specific activator of JNK, blocked the protection of esculetin against H_2_O_2_-induced HepG2 cells apoptosis and pyroptosis. In conclusion, esculetin can protect HepG2 cells against H_2_O_2_-induced oxidative stress, apoptosis, and pyroptosis via inhibiting the JNK signaling pathway. These findings indicate that esculetin has the potential to be used as an antioxidant that improves oxidative stress-related diseases.

## 1. Introduction

The liver is an important organ, which can transform and clean up endogenous metabolites and exogenous toxins, maintaining the homeostasis of the body. It plays a vital role in metabolism, detoxification, bile secretion, and immune defense. However, the liver is also susceptible to exogenous drugs, viruses, alcohol, metabolic disorders, and other factors, leading to various liver diseases [1].

Oxidative stress is caused by an imbalance between the production and clearance of reactive oxygen species (ROS) in the body. The abundant mitochondria that exist in the liver and its vigorous metabolism make it easy for liver cells to accumulate a large amount of ROS. Excessive ROS can result in oxidative damage to biofilm systems, proteins, and DNA, leading to cell death in serious cases, which plays an important role in the pathogenesis of many chronic liver diseases, such as liver injury, hepatitis, cirrhosis, and liver cancer [2]. Numerous studies demonstrated that natural antioxidants, such as quercetin [3] and curcumin [4], are widely used in daily life, and can effectively inhibit ROS-induced oxidative stress, which may help to improve the severity of these liver diseases.

**Figure 1:**
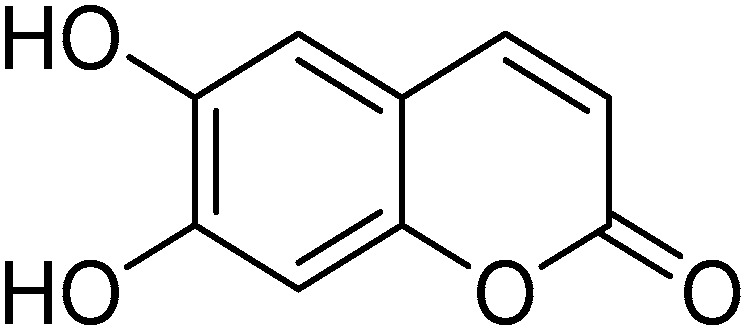
Molecular structure of esculetin.

Esculetin (Esc), a natural coumarin (Figure 1), is a major active ingredient of the traditional Chinese medicine *Cortex Fraxini* [5]. Esc is a natural antioxidant with good antioxidant properties, which possesses a strong free radical-scavenging capacity [6]. Esc can protect ARPE-19 cells and HEK293 cells from t-BHP-induced oxidative stress and apoptosis [7,8]. Esc can also inhibit amyloid protein-induced oxidative stress and neuronal death in SH-SY5Y cells through the activation of Nrf2 and increase in GSH [9]. Esc improves H_2_O_2_, t-BHP or ethanol-induced oxidative damage of liver cells by reducing ROS and malondialdehyde (MDA) production and regulating the redox system through the Nrf2/NQO1 pathway [10,11,12]. Besides, Esc also protects against t-BHP, CCl4, or ethanol-induced liver injury in animal models by reducing oxidative stress and lowering the levels of alanine transaminase (ALT) and aspartate transaminase (AST) in serum [11,12,13]. However, to date, there has been no report regarding whether Esc improves H_2_O_2_-induced oxidative stress in HepG2 cells and thus inhibits mitochondrial apoptosis and pyroptosis.

Therefore, this study aimed to investigate the hepatoprotective effect and mechanism of Esc against H_2_O_2_-induced oxidative damage, apoptosis, and pyroptosis in HepG2 cells by MTT assays, DCFH-DA assays, LDH assays, flow cytometry, and Western blot assays. Our findings will provide a theoretical basis for the application of Esc as an antioxidant to improve oxidative stress.

## 2. Results

### 2.1. Esc Protected HepG2 Cells against H_2_O_2_-Induced Oxidative Stress

**Figure 2:**
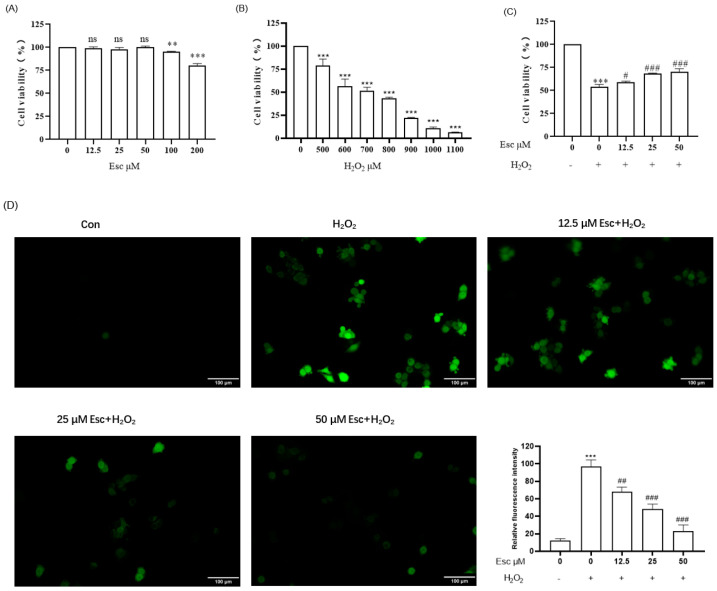
Esc protected HepG2 cells against H_2_O_2_−induced oxidative stress. (**A**) Effect of Esc on the viability of HepG2 cells. (**B**) Effect of H_2_O_2_ on the viability of HepG2 cells. (**C**) Effect of Esc on the viability of H_2_O_2_−induced HepG2 cells. (**D**) Effect of Esc on intracellular ROS levels of H_2_O_2_−induced HepG2 cells (200×, bar scale = 100 μm). All data are presented as mean ± SD (*n* = 3). ** *p* < 0.01, *** *p* < 0.001 vs. Control. ^#^ *p* < 0.05, ^##^ *p* < 0.01, ^###^ *p* < 0.001 vs. H_2_O_2_ group. Con: control; Esc: esculetin.

Firstly, HepG2 cells were treated with different concentrations (0~200 μM) of Esc for 12 h to test the cytotoxicity via MTT assay. As shown in Figure 2A, the viability of HepG2 cells was not affected when the concentration of Esc was at 0~50 μM. Therefore, pretreatment with Esc (12.5, 25, and 50 μM) for 12 h was used for the following experiments. Then, HepG2 cells were exposed to the different concentrations of H_2_O_2_ (0~1100 μM) for 6 h to choose the optimal dose of H_2_O_2_ treatment (Figure 2B). The results revealed that when the concentration of H_2_O_2_ was at 700 μM, the viability of HepG2 cells was 51.54 ± 3.83%. So, 700 μM H_2_O_2_ was used to establish the HepG2 cell oxidative damage model. Next, the protective effect of Esc on H_2_O_2_-induced oxidative damage in HepG2 cells was shown in Figure 2C. The cell viability of the H_2_O_2_ model group decreased to 54.27 ± 2.36% compared with that of the control group. On the contrary, compared with the H_2_O_2_ model group, the cell viability of the Esc (12.5, 25, and 50 μM) group increased to 58.98 ± 1.29%, 68.52 ± 0.45%, and 70.42 ± 3.26% respectively. This indicated that Esc can protect HepG2 cells from H_2_O_2_-induced repression of cell viability in a dose-dependent manner. Finally, the levels of intracellular ROS were tested by the 2’, 7’-dichlorofluorescin diacetate (DCFH-DA) probe. As presented in Figure 2D, compared with the control group, the intracellular ROS level significantly increased after HepG2 cells exposure to 700 μM H_2_O_2_ alone for 6 h, while the production of intracellular ROS was notably reduced by pretreatment with 25 μM or 50 μM Esc. These data suggested that Esc could ameliorate H_2_O_2_-induced oxidative stress in HepG2 cells.

### 2.2. Esc Protected HepG2 Cells from H_2_O_2_-Induced Apoptosis

**Figure 3:**
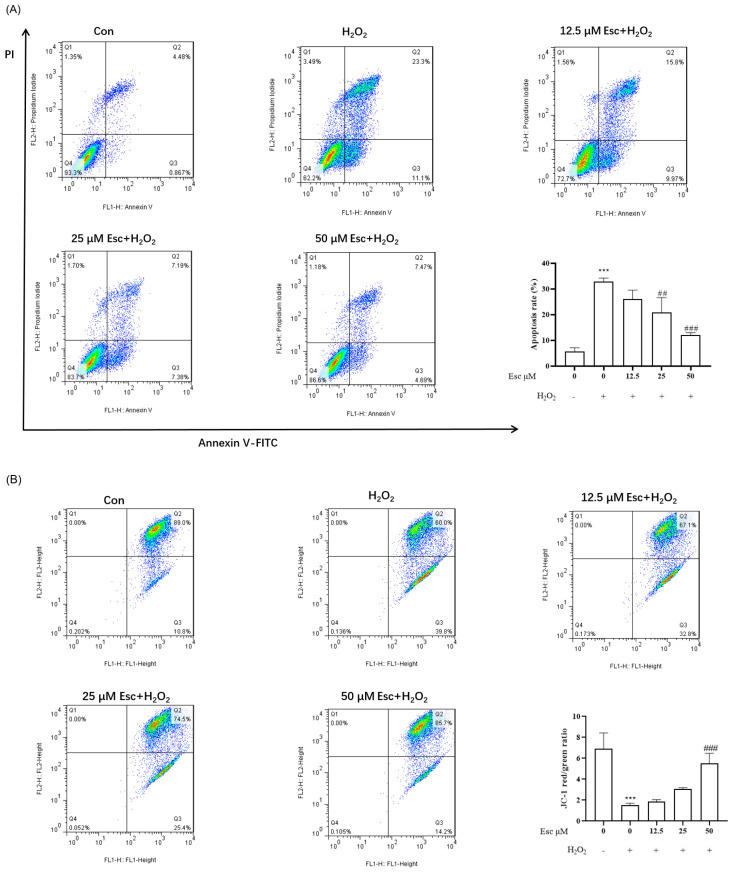

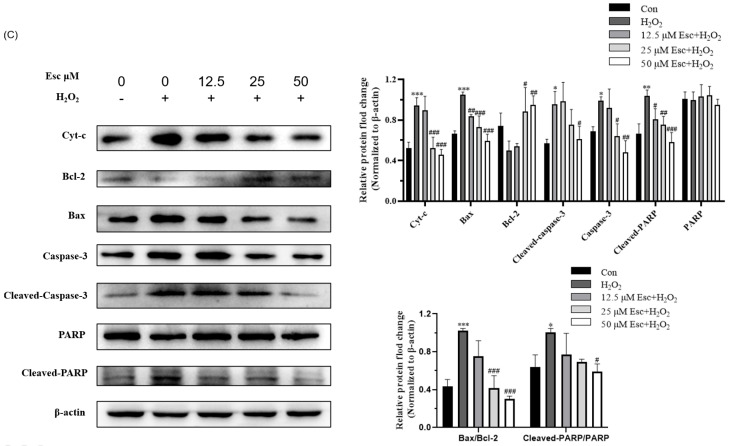
Esc protected HepG2 cells from H_2_O_2_−induced apoptosis. HepG2 cells were exposed to H_2_O_2_ for 6 h with Esc (0, 12.5, 25, and 50 μM) pretreatment for 12 h. (**A**) The apoptosis rate of HepG2 cells was determined by flow cytometry. (**B**) The mitochondrial membrane potential of HepG2 cells was detected by flow cytometry. (**C**) The apoptosis proteins levels in each group were measured by Western blot analysis. All data are presented as mean ± SD (*n* = 3). * *p* < 0.05, ** *p* < 0.01, *** *p* < 0.001 vs. Control. ^#^ *p* < 0.05, ^##^ *p* < 0.01, ^###^ *p* < 0.001 vs. H_2_O_2_ group. Con: control; Esc: esculetin.

We investigated whether Esc plays a cytoprotective effect in HepG2 cells through suppressing cell apoptosis. Firstly, the apoptosis rate of HepG2 cells was tested by flow cytometry. As shown in Figure 3A, with exposure to H_2_O_2_ alone for 6 h, the apoptosis rate was increased to 32.88 ± 1.35%, whereas after pretreatment with 50 μM Esc, the apoptotic rates apparently reduced to 12.14 ± 0.90%. Secondly, mitochondrial membrane potential (MMP) was detected by JC-1 staining. Compared with the control group, the red fluorescence intensity of the H_2_O_2_ mode group was dramatically attenuated, suggesting HepG2 cells exhibited remarkable mitochondrial dysfunction and damage. In contrast, pretreatment with Esc effectively prevented H_2_O_2_-induced MMP loss (Figure 3B). Finally, to further confirm the above results, the expression of apoptosis-related proteins was measured by Western blot analysis. Compared with those in the H_2_O_2_ mode group, Esc pretreatment significantly decreased cleaved-Caspase-3, cleaved-PARP, pro-apoptotic protein (Bax) and MMP-related protein (Cyt-c) expression, and increased anti-apoptotic protein (Bcl-2) expression in HepG2 cells (Figure 3C). Accordingly, the ratio of Bax/Bcl-2 and cleaved-PARP/RAPR was markedly decreased in the presence of Esc (Figure 3C). Taken together, these data confirmed that Esc protected HepG2 cells from H_2_O_2_-induced mitochondrial apoptosis. 

### 2.3. Esc Protected HepG2 Cells from H_2_O_2_-Induced Pyroptosis

**Figure 4:**
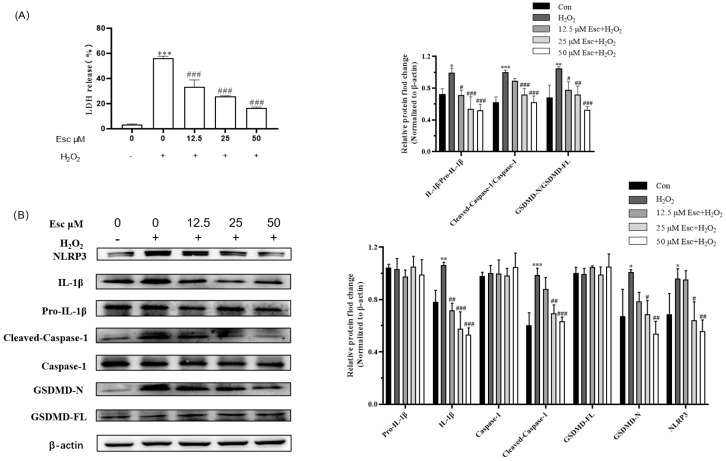
Esc protected HepG2 cells from H_2_O_2_−induced pyroptosis. HepG2 cells were exposed to H_2_O_2_ for 6 h with Esc (0, 12.5, 25, and 50 μM) pretreatment for 12 h. (**A**) The LDH release of HepG2 cells was tested by LDH assay. (**B**) The pyroptosis−related proteins of HepG2 cells were measured by Western blot analysis. All data are presented as mean ± SD (*n* = 3). * *p* < 0.05, ** *p* < 0.01, *** *p* < 0.001 vs. control. ^#^ *p* < 0.05, ^##^ *p* < 0.01, ^###^ *p* < 0.001 vs. H_2_O_2_ group. Con: control; Esc: esculetin.

Furthermore, we investigated whether Esc plays a cytoprotective effect in HepG2 cells through inhibiting cell pyroptosis. LDH assay showed the LDH release of the H_2_O_2_ model group significantly increased to 56.13 ± 1.59%, while pretreatment with 50 μM Esc remarkably reduced the level of LDH to 16.50 ± 0.83% (Figure 4A). Meanwhile, the expression of pyroptosis-related proteins was measured by Western blot analysis. Compared with the H_2_O_2_ mode group, Esc pretreatment significantly decreased the expression of the pyroptosis-related proteins NLRP3, cleaved-Caspase-1, Il-1β, and GSDMD-N (Figure 4B). Accordingly, the ratio of cleaved-Caspase-1/Caspase-1, Il-1β/Pro-Il-1β, and GSDMD-N/GSDMD-FL was markedly decreased in the presence of Esc (Figure 4B). In short, these results suggested that Esc protected HepG2 cells from H_2_O_2_-induced pyroptosis.

### 2.4. Esc Protected HepG2 Cells against H_2_O_2_-Induced Oxidative Stress via the JNK Signaling Pathway

**Figure 5:**
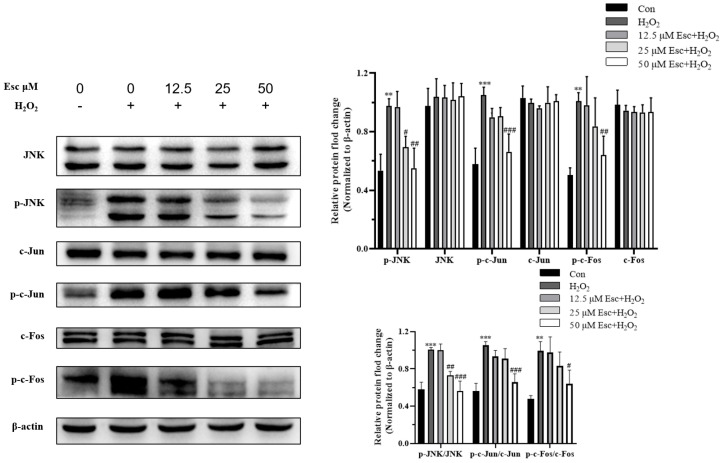
Esc protected HepG2 cells against H_2_O_2_−induced oxidative damage via the JNK signaling pathway. HepG2 cells were exposed to H_2_O_2_ for 6 h with Esc (0, 12.5, 25, and 50 μM) pretreatment for 12 h. The JNK signaling pathway−related proteins of HepG2 cells were measured by Western blot analysis. All data are presented as mean ± SD (*n* = 3). ** *p* < 0.01, *** *p* < 0.001 vs. Control. ^#^ *p* < 0.05 or ^##^ *p* < 0.01 or ^###^ *p* < 0.001 vs. H_2_O_2_ group. Con: control; Esc: esculetin.

To determine whether JNK is involved in Esc protected HepG2 cells against H_2_O_2_-induced oxidative stress, the JNK signaling pathway related proteins were measured by Western blot analysis. As shown in Figure 5, compared with those in the H_2_O_2_ mode group, the protein expression of p-JNK, p-c-Fos, and p-c-Jun was significantly decreased by Esc pretreatment. Accordingly, the ratio of p-JNK/JNK, p-c-Fos/c-Fos, and p-c-Jun/c-Jun was markedly decreased in the presence of Esc (Figure 5). These illustrated that Esc protected HepG2 cells against H_2_O_2_-induced oxidative stress was closely related to the JNK signaling pathway. 

### 2.5. Ani Reversed the Protection of Esc against H_2_O_2_-Induced HepG2 Cells’ Oxidative Stress, Apoptosis, and Pyroptosis

**Figure 6:**
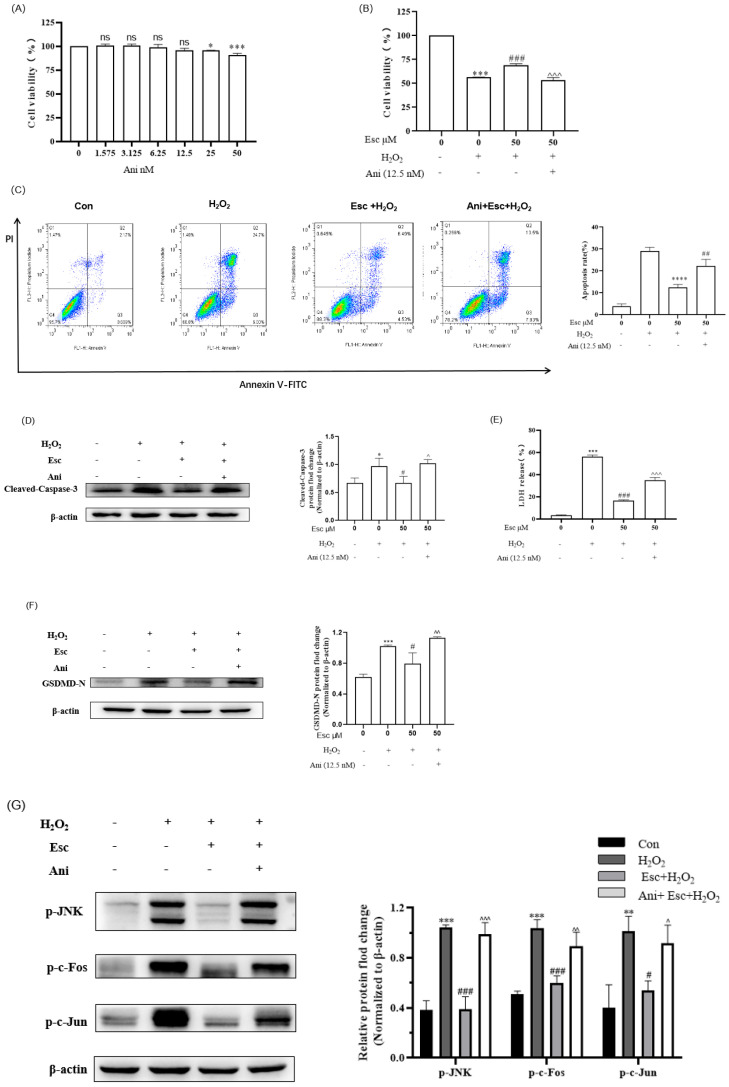
Ani reversed the protection of Esc against H_2_O_2_−induced HepG2 cells’ oxidative stress, apoptosis, and pyroptosis. (**A**) The cytotoxicity of Ani on HepG2 cells. (**B**) The effect of Ani and Esc on the viability of H_2_O_2_−induced HepG2 cells. (**C**) The apoptosis rate of HepG2 cells was determined by flow cytometry. (**D**) The protein expression level of cleaved−Caspase-3 was measured by Western blot analysis. (**E**) LDH release from HepG2 cells was tested by LDH assay. (**F**) The protein expression level of GSDMD-N was measured by Western blot analysis. (**G**) The protein expression levels of p−JNK, p−c−Fos, and p−c−Jun were measured by Western blot analysis. Data are presented as mean ± SD (*n* = 3). * *p* < 0.05, ** *p* < 0.01, *** *p* < 0.001, **** *p* < 0.0001 vs. Control. ^#^ *p* < 0.05, ^##^ *p* < 0.01, ^###^ *p* < 0.001 vs. H_2_O_2_ group. ^ *p* < 0.05, ^^ *p* < 0.01, ^^^ *p* < 0.001 vs. Esc experimental group. Con: control; Esc: esculetin; Ani: anisomycin.

To further verify that the protection of Esc against H_2_O_2_-induced HepG2 cells oxidative stress was correlated with the JNK signaling pathway, the JNK-specific activator anisomycin (Ani) was used for revalidation (Figure 6). As shown in Figure 6A, when the concentration of Ani was at 12.5 nM or below, it had no cytotoxic effect on HepG2 cells, so 12.5 nM Ani was selected for subsequent experiments. Firstly, our results demonstrated that the combined treatment (Ani and Esc) obviously decreased the cell viability of HepG2 compared with the Esc treatment (Figure 6B). Then, the percentage of apoptotic cells (Figure 6C), and the apoptotic protein (cleaved-Caspase-3) expression (Figure 6D) in the combined treatment group (Ani and Esc) was significantly increased compared with that of the Esc treatment group. Likewise, the generation of LDH release (Figure 6E), and the pyroptosis related-protein (GSDMD-N) expression (Figure 6F) in the combined treatment group (Ani and Esc) was also increased. These results indicated that Ani can reverse the protection of Esc against H_2_O_2_-induced HepG2 cells apoptosis and pyroptosis. Moreover, Western blot analysis of the JNK signaling pathway related-proteins showed that the combined treatment group (Ani and Esc) markedly inhibited the downregulation of p-JNK, p-c-Fos, and p-c-Jun expression in H_2_O_2_-induced HepG2 cells compared with the Esc treatment group (Figure 6G). Collectively, Esc can protect HepG2 cells against H_2_O_2_-induced oxidative stress, apoptosis, and pyroptosis by inhibiting the JNK signaling pathway. 

## 3. Discussion

Oxidative stress is a negative factor caused by excessive free radicals in the body, which is crucial in the pathogenesis of many chronic liver diseases, such as liver injury, hepatitis, cirrhosis, nonalcoholic fatty liver disease, and liver cancer. Accumulating evidence has shown that natural antioxidants are commonly used in daily life, playing vital roles in antioxidative stress and thus improving these chronic liver diseases. Esculetin (Esc) is a major active coumarin isolated from the traditional Chinese medicine *Cortex Fraxini* [5], which exhibits notable antioxidant, anti-inflammatory, anti-bacterial, and anti-cancer pharmacological effects [14]. Previous studies have demonstrated that Esc not only improves H_2_O_2_-, t-BHP- or ethanol-induced oxidative damage of liver cells, but also protects t-BHP-, CCl4-, or ethanol-induced liver injury in animal models [10,11,12,13]. However, to date, there has been no report of whether Esc improving H_2_O_2_-induced oxidative stress in HepG2 cells is related to the inhibition of mitochondrial apoptosis and pyroptosis. Therefore, we aimed to investigate the protective function and the mechanism of Esc against H_2_O_2_-induced oxidative stress, apoptosis and pyroptosis in HepG2 cells for the purpose of exploring the potential use of Esc in attenuating oxidative stress.

H_2_O_2_ is the main component of intracellular ROS that are produced during many physiological and pathological processes, which can easily cross the cell membrane, directly destroy the structural stability of DNA and protein, induce lipid peroxidation, and cause oxidative stress [15]. As an ideal inducer, H_2_O_2_ is often used to establish cell oxidative damage models and analyze the mechanism of oxidative stress. In our experiments, when HepG2 cells were exposed to 700 μM H_2_O_2_ for 6 h, the cell viability of HepG2 cells was notably decreased, and the ROS production was significantly accumulated. It was determined that Esc pretreatment can effectively decrease the ROS level and improve the cell viability in a dose-dependent manner. These results confirmed that Esc can alleviate H_2_O_2_-induced oxidative stress in HepG2 cells.

Excessive ROS will cause lipid peroxidation of the mitochondrial membrane, which will lead to opening of the mitochondrial permeability transition pore and will depolarize the mitochondrial membrane. Cyt-c is released from the mitochondria and binds to apoptotic protease activating factor 1 (Apaf-1), triggering the caspase family cascade reaction that initiates the mitochondrial apoptosis pathway [16,17,18]. It was reported that H_2_O_2_ can reduce the mitochondrial membrane potential and increase the apoptotic protein expression of cleaved Caspase-3, thus inducing apoptosis in HepG2 cells. Previous studies indicated that Esc could improve oxidative stress in HEK293 cells and H9c2 cells by suppressing mitochondrial apoptosis pathways [7,19]. These findings implied that Esc improves H_2_O_2-_induced oxidative stress in HepG2 cells, which might be related to inhibition of the mitochondrial apoptosis pathway. Our results revealed that pretreatment with Esc not only apparently increased anti-apoptotic protein (Bcl-2) expression and prevented MMP loss, but also markedly reduced the apoptotic rates and the expression of cleaved-Caspase-3, cleaved-PARP, pro-apoptotic protein (Bax), and MMP-related protein (Cyt-c) in H_2_O_2_-induced HepG2 cells. In short, it can be concluded that Esc can alleviate H_2_O_2_-induced oxidative stress in HepG2 cells by inhibiting mitochondrial apoptosis pathway.

Pyroptosis is initiated by the activation of inflammasomes. The NLRP3 inflammasome triggers the activation of Caspase-1, and cleavage of pro-IL-1β and Gasdermin D (GSDMD), thereby resulting in the formation of membrane openings and the release of inflammatory cytokine (IL-18 and IL-1β), ultimately leads to cell pyroptosis. A large amount of studies confirmed that ROS can cause mitochondrial damage in various liver cells (HL7702, AML12, LO2, LM3, and Huh7) though activating the NLRP3 mediated-pyroptosis pathway [20,21,22]. Recent evidence demonstrated that Esc has good anti-inflammation effects and can treat intestinal inflammatory diseases (IBD) by inhibiting the NLRP3 mediated-pyroptosis pathway [23,24]. Based on this, we speculate that Esc improves H_2_O_2-_induced oxidative stress in HepG2 cells, which might be associated with the NLRP3 mediated-pyroptosis pathway. Our results suggested that pretreatment with Esc remarkably decreased the level of LDH, but also significantly reduced the expression of the pyroptotic proteins NLRP3, cleaved-Caspase-1, Il-1β, and GSDMD-N in H_2_O_2_-induced HepG2 cells. Therefore, these results indicated that Esc alleviates H_2_O_2_-induced oxidative stress in HepG2 by inhibiting the NLRP3 mediated-pyroptosis pathway.

Previous studies have shown that H_2_O_2_ can induce cell apoptosis through the JNK/AP-1 signaling pathway [25,26]. Meanwhile, H_2_O_2_ can also result in cell pyroptosis by activating Caspase-1 and GSDMD proteins [27]. In recent years, it has been reported that Esc can improve oxidative stress and apoptosis in H9c2 cells by reducing the phosphorylation level of JNK in order to attenuate myocardial ischemia/reperfusion (I/R) injury [28]. In addition, Esc can protect oxidative stress-induced aging in human HaCaT keratinocytes by decreasing the phosphorylation level of c-Jun and c-Fos and inhibiting the JNK signaling pathway [29]. Thus, we hypothesized that the protective effect of Esc in H_2_O_2_-induced HepG2 cells might be closely associated with the JNK signaling pathway. Our Western blot results signified that Esc pretreatment evidently downregulated the proteins expression of p-JNK, p-c-Fos, and p-c-Jun. Then, using the JNK specific activator (anisomycin) to reverse verification, it was revealed that anisomycin upregulated the expression of the apoptotic protein (cleaved-Caspase-3), the pyroptosis related-protein (GSDMD-N), and the JNK signaling pathway related-proteins of p-JNK, p-c-Fos, and p-c-Jun. This indicated that anisomycin reversed the protection of Esc in H_2_O_2_-induced HepG2 cells. Taken together, Esc was able to attenuate oxidative stress, apoptosis, and pyroptosis in H_2_O_2_-induced HepG2 cells by inhibiting the JNK signaling pathway.

## 4. Materials and Methods

### 4.1. Reagents and Antibodies

Esculetin was purchased from Yuanyebio (Shanghai, China). Anisomycin (Ani), and methylthiazolyldiphenyl-tetrazolium bromide (MTT) was purchased from Solarbio (Beijing, China). A LDH Detection Kit and a JC-1 Staining Kit were purchased from Beyotime (Shanghai, China). An Annexin V-FITC/PI Apoptosis Detection Kit was purchased from Key gen Biotech (Nanjing, China). DMEM was purchased from Sigma Aldrich (St. Louis, IL, USA). Fetal bovine serum was purchased from ExCell Bio (Taicang, China). Antibodies against Bax, Bcl-2, Caspase-1, and p-c-Jun were purchased from Proteintech (Chicago, MO, USA). Antibodies against cleaved-Caspase-3, and p-c-Fos were purchased from Cell Signaling Technology (Danvers, MA, USA). Antibodies against c-Fos, c-Jun, Caspase-3, JNK, and PARP were purchased from Beyotime (Shanghai, China). Anti-β-actin antibody was purchased from Solarbio (Beijing, China). Antibodies against cleaved-PARP, Cytochrome c (Cyt-c), GSDMD, IL-1β, NLRP3, and p-JNK were purchased from Abcam (Cambridge, UK). 

### 4.2. Cell Culture

HepG2 cells were purchased from the Cell Bank of the Chinese Academy of Sciences (Shanghai, China) and cultured at 37 °C in high-glucose DMEM, supplemented with 10% FBS, 1% 100 unit/mL penicillin and 100 µg/mL streptomycin with 5% CO_2_.

### 4.3. Cell Viability Assay

Cell viability was evaluated by MTT assay. HepG2 cells treated with neither Esc nor H_2_O_2_ were used as the control group. Briefly, HepG2 cells were seeded in a 96-well plate at a density of 1 × 10^4^ cells/100 μL in DMEM medium and cultured for 12 h. Next, the cells were treated with 700 μM H_2_O_2_ for 6 h with or without 12 h pretreatment with different concentrations of Esc or Ani. Then, 10 μL 5 mg/mL MTT solution was added to each well and incubated at 37 ℃ in the dark for 4 h. The liquid was discarded and 150 μL DMSO was added to each well. The absorbance at 490 nm was determined using a microplate reader (Bio-Rad, Hercules, CA, USA). The cell viability was calculated using the following formula:

cell viability (%) = (A_experimental group_ − A_blank group_)/(A_control group_ − A_blank group_) × 100%

### 4.4. ROS Production Assay

The ROS productions in HepG2 cells were tested using the dichloro dihydrofluorescein diacetate (DCFH-DA) Detection Kit method. HepG2 cells were seeded in six-well plates at a density of 2.5 × 10^5^ cells/well and incubated with different concentrations of Esc for 12 h. Next, the cells were exposed to 700 μM H_2_O_2_ for 6 h. Then, the cells were washed twice and incubated with DCFH-DA for 30 min at 37 ℃ in the dark. The ROS relative intensity of cells was observed under a fluorescence microscope (Olympus, Tokyo, Japan). Green fluorescence intensity was measured by imageJ 1.8.0 software.

### 4.5. Apoptosis Rate Assay

Reh Cell apoptosis was tested by using an Annexin-V-FITC Apoptosis Detection Kit. HepG2 cells were seeded in 6-well plates at 2.5 × 10^5^ cells/well and exposed to 700 μM H_2_O_2_ for 6 h, with or without 12 h of pretreatment with different concentrations of Esc. Cells were harvested and double-stained with Annexin V-FITC and PI for 10 min in the dark. The apoptotic rate (%) of HepG2 cells was detected via flow cytometry (Piscataway, NJ, USA).

### 4.6. Mitochondrial Membrane Potential Assay

Mitochondrial membrane potential (MMP) was tested by JC-1 assay. HepG2 cells were seeded in six-well plates at 2.5 × 10^5^ cells/well and exposed to 700 μM H_2_O_2_ for 6 h, with or without 12 h of pretreatment with different concentrations of Esc. After being washed with PBS, the cells were harvested and stained with JC-1 for 40 min in the dark. Then, the cells were washed twice with staining buffer. Changes in the mitochondria of each group were detected via flow cytometry (Piscataway, NJ, USA).

### 4.7. LDH Release Assay

The LDH content in the culture medium was tested with the LDH Release Assay kit. HepG2 cells were seeded in 96-well plates at 1 × 10^4^ cells/well and exposed to 700 μM H_2_O_2_ for 6 h, with or without 12 h of pretreatment with different concentrations of Esc. Then, the LDH release reagents were added to the plate 1 h before the test. After centrifuging, the supernatant was collected and incubated with LDH working solution in the dark for 30 min. The absorbance at 490 nm was determined using a microplate reader.

### 4.8. Western Blot Analysis

The total protein of HepG2 cells was extracted with RIPA buffer (Solarbio) and the protein concentration was quantified using a BCA protein assay kit (ComWin Biotech, Beijing, China). Protein (20 µg) in different groups was separated on 10–15% SDS-PAGE (Beyotime) and transferred to PVDF membrane (Millipore, USA). After being blocked with 5% BSA (Soiarbio) at room temperature for 2 h, membranes were incubated with primary antibodies (Bax, Bcl-2, Caspase-3, cleaved-Caspase-3, PARP, cleaved-PARP, Cyt-c, Caspase-1, GSDMD, IL-1β, Pro-Il-1β, NLRP3, JNK, p-JNK, c-Fos, p-c-Fos, c-Jun, p-c-Jun, and β-actin) overnight at 4 ℃. Subsequently, the membranes were washed with Tris-buffered saline and Tween 20 (TBST) six times and incubated with the corresponding secondary antibodies at room temperature for 1 h. Finally, protein bands were visualized using an ECL developer (New Cell & Molecular Biotech, Suzhou, China) and scanned in a chemiluminescence imager (Tanon, Shanghai, China). The protein levels were calculated relative to that of β-actin.

### 4.9. Statistical Analysis

Data are presented as the mean ± SD. Each experiment was repeated at least three times. GraphPad Prism 8 software was used for data analysis. Statistical analysis was performed by *t*-test or one-way analysis of variance (ANOVA), and a *p* < 0.05 and below was regarded as statistically significant.

## 5. Conclusions

In conclusion, we verified that esculetin could protect HepG2 cells against H_2_O_2_-induced oxidative stress by attenuating apoptosis and pyroptosis by inhibiting the JNK signaling pathway. These results suggest that esculetin has the potential to be used as an antioxidant to improve oxidative stress. In the future, the protective effects and mechanism of esculetin against oxidative stress in animal models will be investigated to provide a theoretical basis for applying Esc to prevent oxidative stress-related diseases.

## Data Availability

The data that support the findings of this study are available from the corresponding author upon reasonable request.
